# Letter to the editor

**DOI:** 10.1186/s13054-021-03635-0

**Published:** 2021-07-20

**Authors:** Folke Sjöberg, Lotti Orwelius, Michelle Chew, Sören Berg, Sten Walther

**Affiliations:** 1 Department of Biomedical and Clinical Sciences, Linköping University, Linköping, Sweden; 2 Department of Anaesthesiology and Intensive Care, Linköping University Hospital, Linköping, Sweden; 3 Department of Hand Surgery, Plastic Surgery, Burns and Intensive Care, The Burn Center, Burns Linköping University Hospital, Linköping University Hospital/Linköping University, 58185 Linköping, Sweden; 4 Department of Cardiothoracic Anesthesia and Intensive Care, Linköping University Hospital, Linköping, Sweden

**Dear Editor,**

We read with interest the paper by Dr. Malmgren and co-workers addressing the issue of health-related quality of life (HRQoL) in former ICU patients [1]. We congratulate the authors for their excellent work, and especially in developing a questionnaire more adapted to depict effects on important areas of HRQoL in ICU patients. There are issues that we want to highlight and comment.

First, often HRQoL investigations in ICU patients lack the use of control groups and the group used is matched for both age and sex. Second, the authors are to be complimented to having addressed two important issues in HRQoL research, that of comorbidity and socio-economic background [2, 3], both in the ICU and Control cohort.

The very interesting finding is that the control group cannot at all be compared with the ICU cohort as both comorbidity and the socio-economic profiles were distinctly different.

Previous studies have demonstrated that decreased HRQoL after ICU care is in large part, due to pre-ICU co-morbidity [4] and socio-economic background [2, 3]. For example, in a study of COPD patients in the ICU matched with hospitalized, non-ICU COPD patients with the same COPD score no difference in HRQoL was seen between groups (ICU/non-ICU) after discharge. In another study the ICU cohort was compared to a general internal medicine hospitalized group not being cared for in ICU and again HRQoL outcomes were comparable.

Finally, what can then be concluded from this study [1] with respect to constructing the questionnaire and what can be said about HRQoL in former ICU patients? The imbalance between comorbidities and socio-economic factors between the ICU and control cohorts diminishes the possibility to make robust conclusions regarding the critical care event itself and its potential effects on HRQoL. This has recently been discussed in a Commentary in this journal [5].

This suggests that the findings (decreased HRQoL) in the ICU cohort, is not primarily related to the critical illness period, but rather stems from comorbidity and socio-economic reasons already present before the ICU-period [2].

General ICU cohorts are heterogenous and it is technically difficult to properly adjust for co-morbidities and the socio-economic background. A consequence may be, that we should examine subgroups with specific co-morbidities and socio-economic background and compare the ones being cared for in the ICU with those with the same level of comorbidity and socio-economic background, hospitalized but not in ICU.

## Data Availability

Not applicable.
