# Supplementary material for: Letter to the editor
Source: Crit Care. 2021 Jul 20;25:255. doi: 10.1186/s13054-021-03635-0 (PMC8290535; doi:10.1186/s13054-021-03635-0)
Supplement: Related texts

# Reply: Authors’ response

**Authors:** Johan Malmgren, Ann-Charlotte Waldenström, Elias Johannesson, Christian Rylander, Stefan Lundin

**Affiliations:** 5 grid.8761.8 0000 0000 9919 9582 Department of Anaesthesiology and Intensive Care Medicine, Institute of Clinical Sciences, Sahlgrenska Academy, University of Gothenburg and Sahlgrenska University Hospital, 413 45 Gothenburg, Sweden; 6 grid.8761.8 0000 0000 9919 9582 Department of Oncology, Institute of Clinical Sciences, Sahlgrenska Academy, University of Gothenburg and Sahlgrenska University Hospital, 413 45 Gothenburg, Sweden; 7 grid.412716.7 0000 0000 8970 3706 Department of Social and Behavioural Studies, University West, Trollhättan, Sweden

**Dear Editor,**

We thank Sjöberg et al. for their comments and encouragement regarding our recently published study on developing a long-term follow-up instrument specifically for ICU survivors.

They argue that long-term sequelae after intensive care could mainly be explained by pre-existing comorbidities and/or socioeconomic status instead of being an effect of intensive care. Consequently, a comparison with a non-ICU-treated control group would be pointless. In statistical terms, this can be analysed by implementing a moderation effect of comorbidity and socioeconomic status respectively on the relationship between cohort type and each HRQoL question. By doing this, any asymmetry between the two groups regarding comorbidities and socioeconomic factors can be addressed, and if the authors were to be correct, the interaction effect would emerge and would be quantified as significant. However, before analyses have been performed, no such conclusions can be drawn.

Notably, SF-36 is used in all referred studies. Beautifully described by Lim et al. [6], SF-36 contains very few issues with importance to ICU survivors and may add to the difficulties of adequately capturing differences between ICU survivors and various control groups. We disagree that further subgroup analyses would help. Rather, the Gordian knot might be to obtain pre-ICU data from intensive care patients without using surrogates. This, however, requires a proper measurement tool.
